# Direct and Pollinator-Mediated Effects of Herbivory on Strawberry and the Potential for Improved Resistance

**DOI:** 10.3389/fpls.2017.00823

**Published:** 2017-05-18

**Authors:** Anne Muola, Daniela Weber, Lisa E. Malm, Paul A. Egan, Robert Glinwood, Amy L. Parachnowitsch, Johan A. Stenberg

**Affiliations:** ^1^Environmental and Marine Biology, Åbo Akademi UniversityTurku, Finland; ^2^Department of Ecology, Swedish University of Agricultural SciencesUppsala, Sweden; ^3^Department of Plant Protection Biology, Swedish University of Agricultural SciencesAlnarp, Sweden; ^4^Department of Crop Production Ecology, Swedish University of Agricultural SciencesUppsala, Sweden; ^5^Department of Plant Ecology and Evolution, Evolutionary Biology Centre, Uppsala UniversityUppsala, Sweden

**Keywords:** crop wild relative, diffuse interaction, ecosystem service, *Galerucella tenella*, *Galerucella sagittariae*, florivory, integrated pest management, integrated pest and pollinator management

## Abstract

The global decline in pollinators has partly been blamed on pesticides, leading some to propose pesticide-free farming as an option to improve pollination. However, herbivores are likely to be more prevalent in pesticide-free environments, requiring knowledge of their effects on pollinators, and alternative crop protection strategies to mitigate any potential pollination reduction. Strawberry leaf beetles (SLB) *Galerucella* spp. are important strawberry pests in Northern Europe and Russia. Given that SLB attack both leaf and flower tissue, we hypothesized pollinators would discriminate against SLB-damaged strawberry plants (*Fragaria vesca*, cultivar ‘Rügen’), leading to lower pollination success and yield. In addition we screened the most common commercial cultivar ‘Rügen’ and wild Swedish *F. vesca* genotypes for SLB resistance to assess the potential for inverse breeding to restore high SLB resistance in cultivated strawberry. Behavioral observations in a controlled experiment revealed that the local pollinator fauna avoided strawberry flowers with SLB-damaged petals. Low pollination, in turn, resulted in smaller more deformed fruits. Furthermore, SLB-damaged flowers produced smaller fruits even when they were hand pollinated, showing herbivore damage also had direct effects on yield, independent of indirect effects on pollination. We found variable resistance in wild woodland strawberry to SLB and more resistant plant genotypes than the cultivar ‘Rügen’ were identified. Efficient integrated pest management strategies should be employed to mitigate both direct and indirect effects of herbivory for cultivated strawberry, including high intrinsic plant resistance.

## Introduction

Insect pollination is a crucial ecosystem service that many crops are totally, or partially, dependent on (Ollerton et al., 2011; Kleijn et al., 2015). The global decline in pollinator densities has caused concerns among growers, meaning that action for pollinator rehabilitation is required. Recent studies suggest that modern pesticides are, at least partly, responsible for the pollinator declines, as well as for reduced pollination services (Stanley et al., 2015), prompting ecologists and policy makers to call for reduced use of harmful pesticides (Godfray et al., 2015; Rundlöf et al., 2015; Dicks et al., 2016). Indeed, the abundance of some flower-visiting insects may increase after transition to organic farming (Jonason et al., 2011; Andersson et al., 2012). For example, potted strawberry plants experimentally introduced to organic farms experienced higher pollination than plants introduced to conventional farms (Andersson et al., 2012). Such short-term experimental studies do not, however, acknowledge the higher herbivore densities that often develop in organic plantations and may potentially interfere with pollination. Therefore, the presence of pollinators may not automatically translate into sufficient pollination success, if herbivores are also present.

Studies of both wild plants and domesticated crops suggest that plant reproduction can be disrupted by either foliar or floral herbivory (Strauss, 1997; Strauss and Murch, 2004; McCall and Irwin, 2006; Bronstein et al., 2007; Hladun and Adler, 2009). Herbivores are known to affect plant reproduction both directly and indirectly. Direct effects of herbivory result from consumption of plant tissues which, in turn, may cause resource limitation (Strauss et al., 2002). In addition, given the intrinsic link between plant reproduction and defense mechanisms, direct effects of herbivory may also be mediated via trade-offs between investing in reproduction or investing in defenses (Herms and Mattson, 1992; Strauss et al., 2002). Indirect effects of herbivory are due to pollination limitation that can be caused, for instance, if herbivory corrupts the visual and olfactory signals that plants use to attract pollinators (Lucas-Barbosa et al., 2015; Tsuji et al., 2016). Both direct and indirect effects of herbivory on plant reproduction in crops are likely if pesticide reduction leads to increased herbivory. If yield reductions are directly due to damage to plant tissues, then improvements in yield would require the damaging agent to be controlled (for instance via resistance breeding). If reductions in reproduction are, instead, the result of indirect interactions with pollinators, then increasing yield after damage necessitates optimizing more complex plant-herbivore-pollinator relationships. Therefore investigations of how herbivory affects pollinators, pollination services, and yield are necessary to inform agricultural practices for effective insect management strategies in domesticated, insect-pollinated crops. Here we use woodland strawberry (*Fragaria vesca* cultivar ‘Rügen’) as a model crop to investigate the effect of strawberry leaf beetle [hereafter strawberry leaf beetles (SLB)] herbivory on pollinator behavior, pollination success, and yield.

**FIGURE 1:**
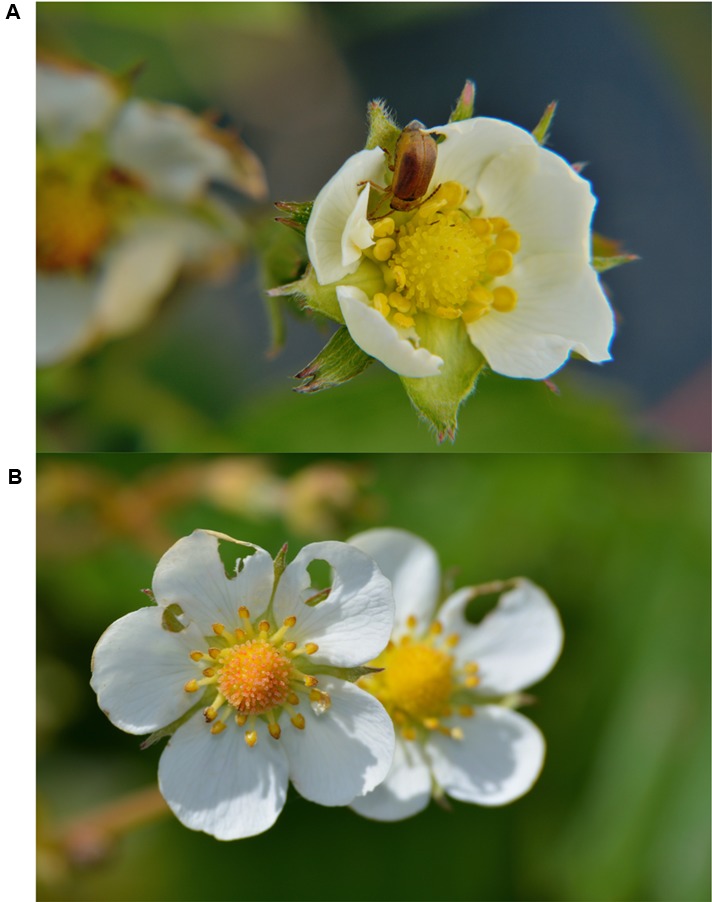
**(A)** Adult strawberry leaf beetle (*Galerucella tenella*) feeding on the petals of a woodland strawberry (*Fragaria vesca*). **(B)** *F. vesca* flowers damaged by *G. tenella*. Photos by Alejandro Ruete.

Garden strawberries suffering from poor insect pollination are known to produce smaller fruits with more deformations and shorter shelf life, reducing their economic value (Andersson et al., 2012; Klatt et al., 2014). Organic and low-pesticide plantations of strawberries in Scandinavia are known to experience outbreaks of SLB of the genus *Galerucella* (Coleoptera: Chrysomelidae), causing damage to both leaves and flowers (**Figure 1**) (Olofsson and Pettersson, 1992). Furthermore, the main damage by univoltine SLB is to early season flowers that produce the most economically valuable fruit. Thus, herbivore-mediated reduction in pollination could reduce yields even if the direct effect of herbivory is low.

One possible intervention to mitigate herbivory-mediated pollination deficiency is to breed for increased resistance, thereby reducing the direct costs of herbivory and indirectly improving pollination. However, plant resistance traits, such as anti-herbivore defenses, have been lost from many crops through breeding during the 20th century (Rodriguez-Saona et al., 2011; Chen et al., 2015; Stenberg et al., 2015). In some cases, however, higher resistance can still be found in the crop’s wild relatives than in current commercial varieties. In such cases high resistance can be restored in future varieties by including wild plant germplasm in breeding programs; this is known as ‘rewilding’ or ‘inverse breeding’ (Andersen et al., 2015; Palmgren et al., 2015).

In this study we investigate whether SLB-damage has both direct and indirect effects on the commercial woodland strawberry cultivar ‘Rügen.’ Specifically, we test if naturally occurring insect pollinators discriminate against SLB-damaged flowers leading to reduced pollination success. Furthermore, we test whether the ability of flowers to produce high-quality fruit is affected by herbivore damage itself or by the activity of pollinators following herbivore damage. We screen wild woodland strawberry genotypes for SLB resistance, and compare their resistance level to the most common commercial cultivar ‘Rügen’ in order to investigate the potential for ‘rewilding’ of strawberry. We hypothesize that wild woodland strawberries sampled from SLB’s native area in Sweden have higher resistance to SLB compared to the commercial cultivar ‘Rügen.’

## Materials and Methods

### Study Species

Strawberry leaf beetles outbreaks with severe effects on strawberry plantations have mainly been reported from Scandinavia (Olofsson and Pettersson, 1992; Stenberg and Axelsson, 2008; Stenberg, 2012, 2014), the Baltic states (Kaufmane and Libek, 2000), and Russia (Bulukhto and Tsipirig, 2004). Two SLB species occur in plantations: *Galerucella tenella* is more common in the south, and *G. sagittariae* is more prevalent in the northern part of Scandinavia. The two SLB species have similar life cycles and cause similar damage to the plant. The overwintered adult beetles usually emerge in April to May. Eggs are laid on the leaves in late May to early June and larvae are found, mainly on the green leaves, in June and July (Olofsson and Pettersson, 1992). Adult beetles forage on both leaves and flowers and can eat petals, seeds, and fruits (**Figure 1**). Larvae mainly feed on the leaves, but are also found on flowers. All *G. sagittariae* and *G. tenella* individuals used in this study were collected from local populations around Uppsala, Sweden.

The German woodland strawberry (*F. vesca*) cultivar ‘Rügen,’ was used for all experiments involving pollinators in this study. Rügen is everbearing, producing flowers throughout the summer until October. In common with most strawberries, ‘Rügen’ has a limited ability for self-pollination, and insects are necessary to pollinate all pistils in a flower (Evans and Jones, 1967). Experimental ‘Rügen’ plantlets for this study were produced by *Mälarö Odling AB* (Ekerö, Sweden).

### Pollinator Response to Manually Damaged Flowers

To determine whether naturally occurring pollinators discriminate between damaged and undamaged flowers, we conducted an observational study where the number of pollinator visits to damaged and undamaged flowers was recorded. Thirty two ‘Rügen’ strawberry individuals were replanted into 2-liter pots containing Hasselfors^TM^ (Hasselfors, Örebro, Sweden) planting soil in early June. All experimental plants were placed outside, on tables in a caged area (30 m × 25 m × 5 m; concrete floor covered on all sides with a metallic mesh; mesh diameter: 20 mm × 20 mm) at the SLU Ultuna campus (N 59° 49.025′, E 17° 39.451′). Temperatures and weather conditions in the caged area follow outside temperatures and conditions. Wild pollinating insects can enter the area, but the mesh excludes birds that could consume the ripened fruits. For each plant individual, half of the open flowers were manually damaged to mimic the damage caused by SLB larvae (see **Figure 1**), and half were left undamaged. Manual damage was conducted by perforating the center of one petal with a pencil which resulted in a hole approximately 3 mm in diameter. Pollinator observations started immediately after damage (8 a.m.) and lasted as long as the pollinators were flying (6 p.m.). We calculated the number of pollinator visits to undamaged control and manually damaged flowers. Flowers were visited by hoverflies (*Syrphidae*) and bees (superfamily *Apoidea*). Due to unfavorable weather conditions during the time that the experimental plants were flowering, the overall number of observed pollinators was low. The study was conducted on July 30, 2012.

### Effects of Herbivory on Pollination Success and Fruit Development

To investigate the effects of herbivory on pollination success and fruit development we used 32 flowering ‘Rügen’ plants (different individuals than in the previous experiment) that were replanted and placed in a caged area, as in the previous experiment. Two flowers that were just about to open were marked for each treatment on each plant (altogether six flowers per plant). During the experiment there were no more than 1–2 additional open flowers per plant individual that were not included in the treatments. Each pair of flowers was randomly assigned to one of the following three treatments: (1) control treatment with undamaged, open pollinated flowers; (2) herbivory treatment with one *G. sagittariae* larva and open pollinated flowers; and (3) herbivory treatment with one *G. sagittariae* larva and hand pollinated flowers. The treatments were designed to measure whether the ability of flowers to produce high-quality fruit is adversely affected by herbivore damage itself (direct costs of herbivory) or by the activity of pollinators following herbivore damage (indirect costs of herbivory). The following procedure was applied to the flowers assigned to the two herbivory treatments (2 and 3): one second instar *G. sagittariae* larva was placed on one petal on each of the two selected flowers per plant. The larvae were removed after 24 h, when they had consumed approximately 50% of the petal area and were still on their experimental flowers. Although we cannot totally exclude the possibility that larvae themselves repelled pollinators, the potential effect on pollination success should be very small. The larvae were present only for 24 h from the beginning of flowering, which typically lasts for about 1 week. For plants in treatment 3, hand pollination was conducted with a marten-hair brush with pollen taken from both the flower’s own anthers and anthers of flowers from three other ‘Rügen’ individuals to mimic pollination by insects. After the initial hand pollination, flowers were checked after 24 and 48 h to ensure a pollination success of at least 80% of pistils successfully pollinated. Hand pollination of herbivore-damaged flowers was undertaken to determine whether the ability of flowers to set fruit is affected by herbivore damage itself or whether it is due to the activity of pollinators.

Pollination success was measured by estimating the percent of successfully pollinated pistils on each flower after blooming. The pistils become darker after successful pollination, allowing estimation of the percentage that have been successfully pollinated. This gives a comparable value of pollination success directly after blooming.

Fruit development was measured in terms of (1) fruit weight and (2) number of deformations. We scored the experimental flowers every second day until all fruits had ripened (about 2 weeks), then weighed the ripe fruits and counted the number of deformations per fruit. Deformations are formed when all pistils are not successfully pollinated, i.e., the fruit body does not swell up around an unfertilised seed (Andersson et al., 2012).

### Resistance of Wild Woodland Strawberry to SLB

To assess the potential for rewilding of strawberry for improved resistance to SLB, we conducted two feeding experiments using 20 randomly chosen wild woodland strawberry genotypes and both *G. tenella* and *G. sagittariae*. To avoid confounding the detection of genetic variation in plant resistance with the potential local adaptation of herbivores, herbivores used in the feeding experiments were collected from other locations than the plants used in these experiments. Furthermore, the collection sites of herbivores were selected so that no *F. vesca* was present at close proximity and, thus, the herbivores were feeding on other species than *F. vesca* (see below for more details). The wild woodland strawberry genotypes were collected in spring 2012 from 20 geographically distinct locations across Uppsala County, Sweden (for geographic coordinates see Supplementary Table S1). Uppsala County is 8207 km^2^ in area, and the 20 locations were randomly selected within this area. The distances between the sampled wild strawberry genotypes varied between 7 and 40 km. At the SLU Ultuna campus, the plant genotypes were cloned from runners for several vegetative generations. In autumn 2013, 40 runners per wild plant genotype were planted randomly in blocks in sandy soil in an open agricultural field in Krusenberg (N 59.741°, E 17.684°), 15 km south of Uppsala. In addition, 40 small ‘Rügen’ plants were randomly planted in the same blocks. The distance between the plants was 50 cm and the entire common garden was covered with fabric mulch (Weibulls Horto) to reduce weed densities. No irrigation or fertilizer was used. The plants were growing in the common garden for 2 years before they were used in this study.

#### Plant Resistance to *Galerucella sagittariae*

We used runners from the common garden in Krusenberg to produce ten genetically identical replicates of each of the 20 woodland strawberry genotypes. Runners were potted in 0.2 L pots containing Hasselfors^TM^ (Hasselfors, Örebro, Sweden) planting soil in early May 2015, and kept in a greenhouse (day 20°C, night 15°C) for 1 month. In early June 2015, pots were placed outside in the caged area and continued to grow for 3 weeks. Eggs of *G. sagittariae* were collected from natural population close to Uppsala (N 59.780°, E 17.753°). We collected *Comarum palustre* cuttings containing *G. sagittariae* eggs, kept them in water at room temperature and checked them daily until larvae hatched. Hatched larvae were allowed to feed on *C. palustre* leaves for 2 days, after which we randomly bagged four larvae on each experimental strawberry plant. Larvae were allowed to feed for 3 weeks. We measured plant size in terms of number of leaves before and after the experiment and calculated the number of damaged leaves at the end of the experiment. Plant resistance was measured as the inverse of plant damage (proportion of damaged leaves) as suggested by Stenberg and Muola (2017).

#### Plant Resistance to *Galerucella tenella*

Adult *G. tenella* were collected from a natural population close to Uppsala (N59.810°, E 17.667°) in early May 2015. The collected beetles were placed in cages in a greenhouse (15°C, LD 16:8 h photoperiod, 80% RH) containing wild strawberry plants of several different, randomly chosen genotypes. The beetles were allowed to mate and oviposit freely in the cages for 24 h, then the plants with eggs were removed. Within 24 h after hatching the larvae were removed from the plants and placed individually in 30 ml plastic containers. The rearing containers with hatched larvae were kept in a climate chamber (15°C, LD 16:8 h photoperiod, 80% RH). Each larva was randomly assigned to 1 of the 19 wild genotypes or to ‘Rügen.’ Ten replicates (=10 larvae) were used for each plant genotype giving a total of 210 rearing containers. The larvae were fed with detached intermediate-aged (fully developed), undamaged leaves obtained from the common garden in Krusenberg. The leaves were exchanged every third day and the rearing containers were always cleaned at the time of leaf exchange. The larvae were checked daily and the pupation date was noted for each larva. Larval development time (days to pupation) was used as a measure of plant resistance to *G. tenella*. In *G. tenella*, larval development time on a host plant correlates positively with egg laying rate and food consumption rate of the adults; thus larval development time is a useful proxy for plant resistance to *G. tenella* (Stenberg et al., 2006).

Our rationale for using the inverse of foliar damage and inverse of herbivore performance when feeding on leaves as proxies for plant resistance arises from the feeding habits of SLB. Ovipositing females and young larvae feed mainly on leaf tissue because flowers normally appear a couple weeks after egg hatching (Olofsson and Pettersson, 1992). Adults and larvae are mobile and thus, they are able to utilize the flowers in their late stage of development. As a result – and especially when density of SLB is high during outbreaks – flowers also get damaged.

### Statistics

Due to the unfavorable weather conditions during the time when experimental plants were flowering and, thus, the short duration of the pollinator observation experiment, the number of observed pollinators was relatively low (number of observed pollinator individuals *n* = 22, of which 17 were hoverflies and 5 bees; total number of flower visits *n* = 120). Thus, to test whether pollinators preferred undamaged control flowers over manually damaged flowers we conducted a non-parametric Sign-test (PROC UNIVARIATE). In addition, we conducted Sign-tests for the two observed pollinator groups (hoverflies and bees) separately in order to test whether these groups behaved similarly, i.e., preferred undamaged control flowers over manually damaged flowers.

To test whether the percentage of successfully pollinated pistils on (1) undamaged, open pollinated control flowers, and (2) *Galerucella sagittariae* damaged, open pollinated flowers differed, we conducted a paired *t*-test. Hand pollinated *G.* ***s****agittariae* damaged flowers were not included in this test since they were repeatedly hand pollinated until they reached at least 80% of successfully pollinated pistils on each flower. In the analysis we used the average number of successfully pollinated pistils in the two flowers per individual plant included in each treatment.

The effect of treatment on fruit weight and the number of deformations was examined using covariance analysis where treatment [(1) undamaged and open pollinated control flowers, (2) herbivore damage and open pollinated flowers, (3) herbivore damage and hand pollinated flowers] was used as a fixed factor. Because garden strawberries that suffer from poor insect pollination are known to produce smaller fruits with more deformations, the effect of pollination success on fruit weight and the number of deformations was controlled by using the percentage of successfully pollinated pistils as a covariate. A statistically significant interaction between treatment and covariate would indicate that the effect of pollination success differs between treatments. However, the interaction was non-significant, so it was removed from the final model. In addition, plant individual was specified as a subject in the repeated statement in order to consider different plants as independent observations instead of each flower (i.e., assuming independence across the subjects). We used a general linear model (PROC MIXED) for fruit weight and a generalized linear model (PROC GLIMMIX) with Poisson error structure for the number of deformations. We used the average fruit weight, the average number of deformations and the average number of successfully pollinated pistils in the two flowers per plant individual included in each treatment in all analyses described above. For hand pollinated flowers we used 80% as an estimate of successfully pollinated pistils in the analysis described above. The normality and equality of variances of the residuals were assessed by visual examination and Levene’s test, respectively.

We conducted a linear mixed model analysis (PROC MIXED) to test whether there is genetic variation in plant resistance (proportion of damaged leaves) to *G. sagittariae* in wild woodland strawberries. Plant size (number of leaves) at the start of the experiment was included as a covariate to account for any potential effect of plant size on larval feeding, and plant genotype and the plant genotype by plant size interaction were included as random factors in the model. Genetic variation in plant resistance to *G. tenella* was examined using one-way ANOVA (PROC MIXED). Here, we used the development time from egg hatching until adulthood as a proxy for plant resistance and plant genotype as a fixed factor in the model. Then, a Tukey’s *post hoc* analysis was conducted to compare each wild genotype with ‘Rügen’; with the commercial cultivar used as a control. This resulted in 19 individual tests, which were corrected for multiple comparisons by the Benjamini and Hochberg method of *p*-value adjustment. Plant genotype was treated differently in the two analyses testing the genetic variation in plant resistance to SLB because in the first model (genetic variation to *G. sagittariae*) ‘Rügen’ cultivar were not included in the experiment and, thus, our aim was to explore the existence of overall genetic variation in plant resistance in wild woodland strawberry population. In the experiment measuring genetic variation to *G. tenella*, the ‘Rügen’ cultivar was included in the experiment and, thus, we were able to compare whether the variation found in the 19 wild woodland strawberry genotypes differed significantly from that observed for ‘Rügen.’ For both analyses, the normality and equality of variances of the residuals was assessed by visual examination and Levene’s test, respectively. All analysis were conducted in SAS (SAS Enterprise Guide 6.1/SAS 9.4, Cary, NC, United States).

## Results

**FIGURE 2:**
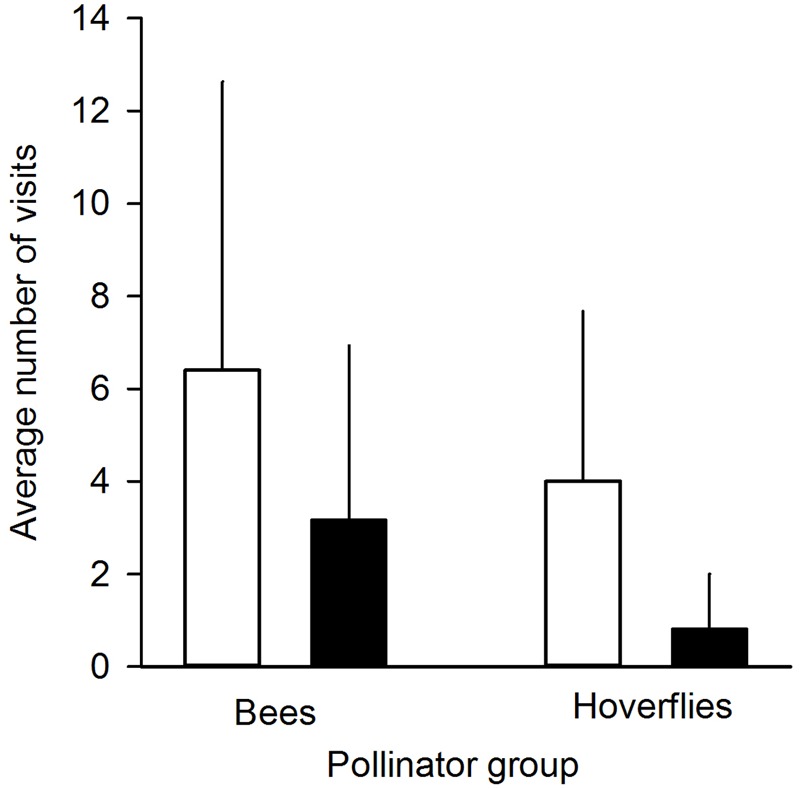
**Average number of pollinator visits to undamaged control flowers (white bars) and manually damaged flowers (black bars).** Pollinators were divided into two groups: bees (*n* = 5 individuals) and hoverflies (*n* = 17 individuals). Mean + SD.

**FIGURE 3:**
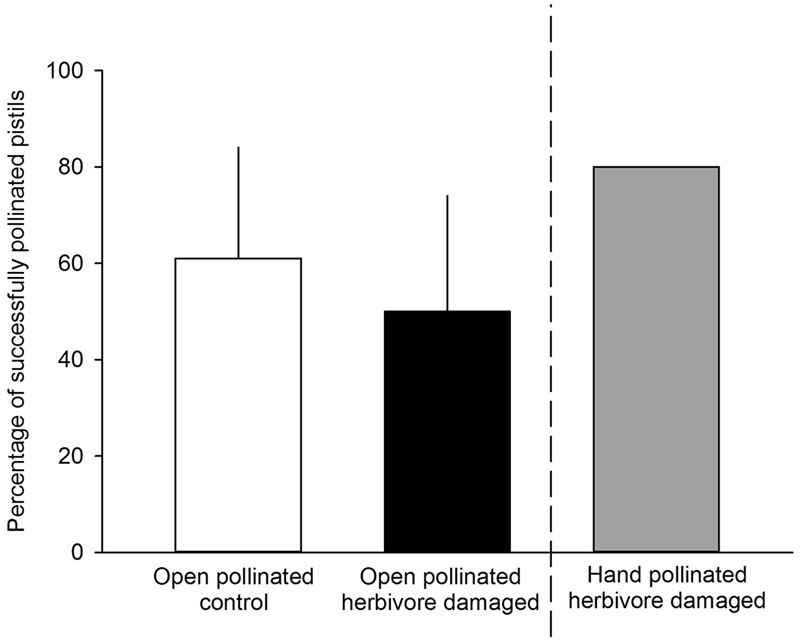
**Percentage of successfully pollinated pistils for either undamaged, open pollinated control flowers (white bars) or *G. sagittariae* damaged, open pollinated flowers (black bars).** *G. sagittariae* damaged hand pollinated flowers were repeatedly hand pollinated until they reached 80% successfully pollinated pistils on each flower (gray bar), and, thus were not included in the analysis. Mean + SD.

We found that herbivory affected pollination in ‘Rügen’ strawberries. Approximately 82% of observed pollinators preferred undamaged control flowers over manually damaged flowers (Sign *M* = 7.5, *P* = 0.0015). Undamaged control flowers were visited 2.5 times more frequently compared to manually damaged flowers (**Figure 2**). However, there was a difference in the preference between the two main pollinator groups. Hoverflies were found to prefer undamaged control flowers over manually damaged flowers (Sign *M* = 5.5, *P* = 0.0127), but there was no significant difference in the preference of bees (Sign *M* = 2, *P* = 0.1250). However, a similar trend was seen in bees; the low number of observed bee pollinators led to less powerful statistical tests which may explain the differences between the groups. Moreover, larval damage by *G. sagittariae* placed on petals significantly decreased the pollination success of ‘Rügen’ strawberries in open pollinated plants (*t* = 2.13, *d.f.* = 31, *P* = 0.041; **Figure 3**): the percentage of successfully pollinated pistils was on average 18% lower on *G. sagittariae* damaged flowers compared to open pollinated undamaged control flowers (**Figure 3**).

**FIGURE 4:**
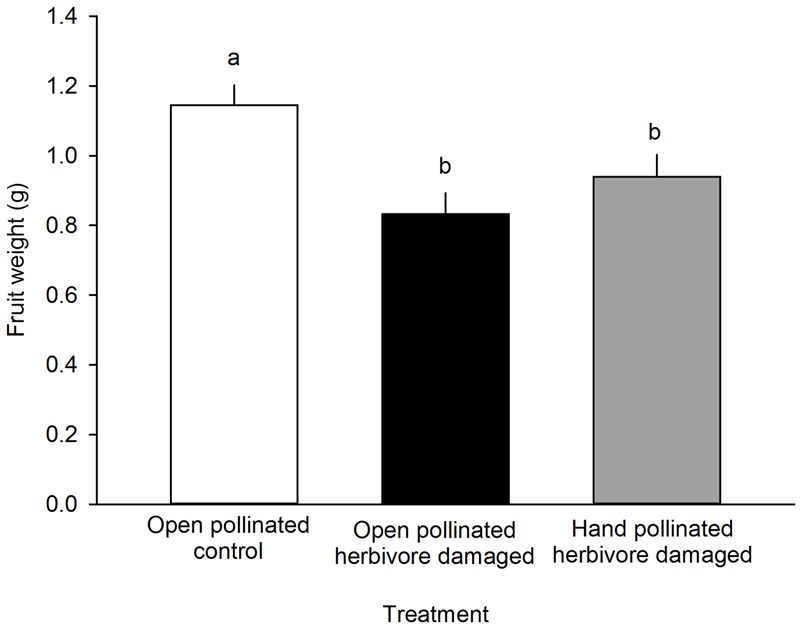
**The effect of treatment on the fruit weight (g) of *F. vesca*.** Different letters indicate statistically significant differences in fruit weight (Tukey’s test) among the different treatments (*P* < 0.05). LS means +SE.

**FIGURE 5:**
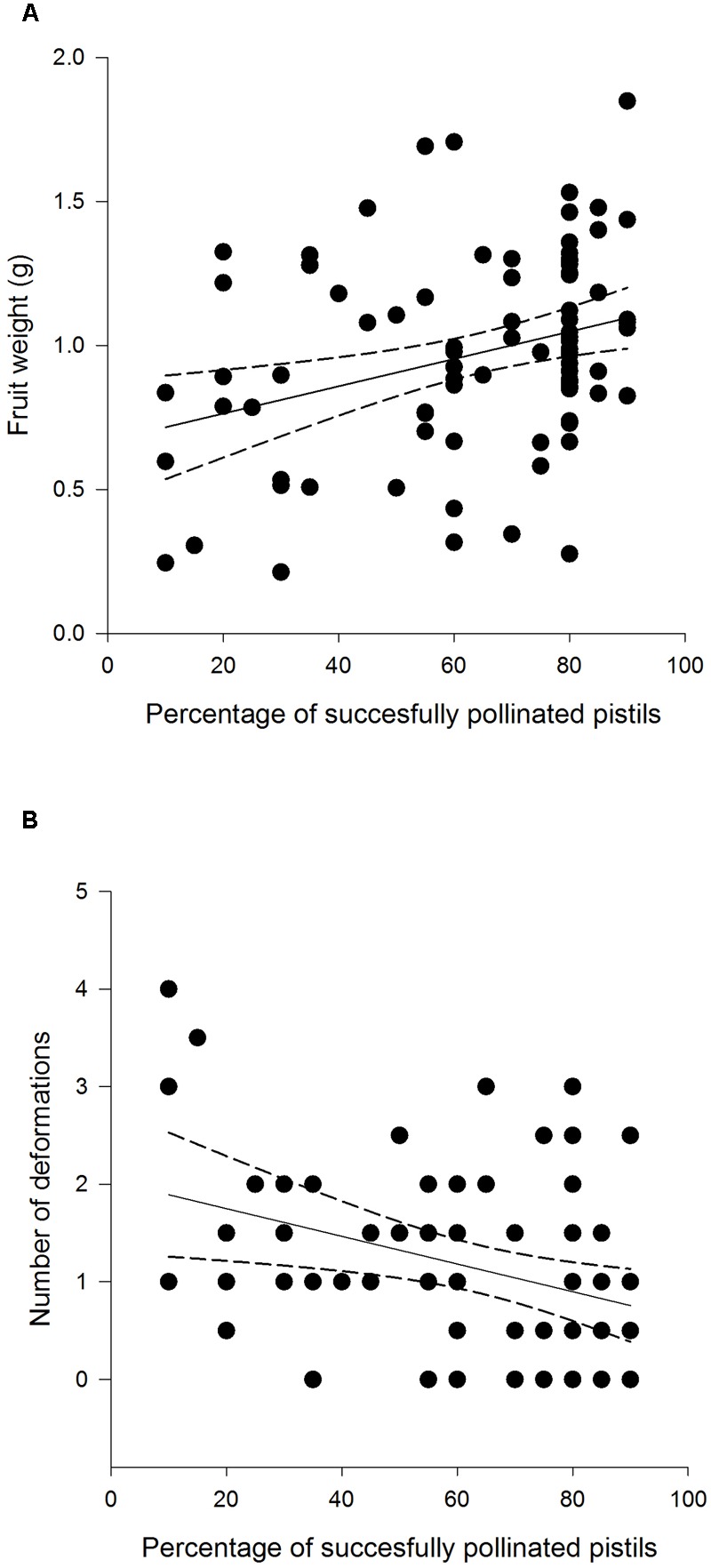
**Weight (A)** and deformation count **(B)** of *F. vesca* fruits produced from flowers that experienced varying degrees of pollination success.

Fruit weight differed among different treatment groups: undamaged, open pollinated flowers set, on average, 0.31 g heavier fruits compared to *G. sagittariae* damaged flowers and 0.21 g heavier fruits compared to *G. sagittariae* damaged hand pollinated flowers (*F* = 7.93, *d.f.* = 2, 83, *P* = 0.0007; **Figure 4**). Fruits had, on average, 1.1 ± 0.9 deformations (mean ± SD). The number of deformations did not differ among the different treatment groups (*F* = 0.59, *d.f.* = 2, 83, *P* = 0.5567). Pollination success (i.e., the percentage of successfully pollinated pistils) affected both fruit weight (*F* = 6.01, *d.f.* = 1, 83, *P* = 0.0163) and the number of deformations (*F* = 6.79, *d.f.* = 1, 83, *P* = 0.0109): higher pollination success resulted in heavier and less deformed fruits (**Figure 5**).

**FIGURE 6:**
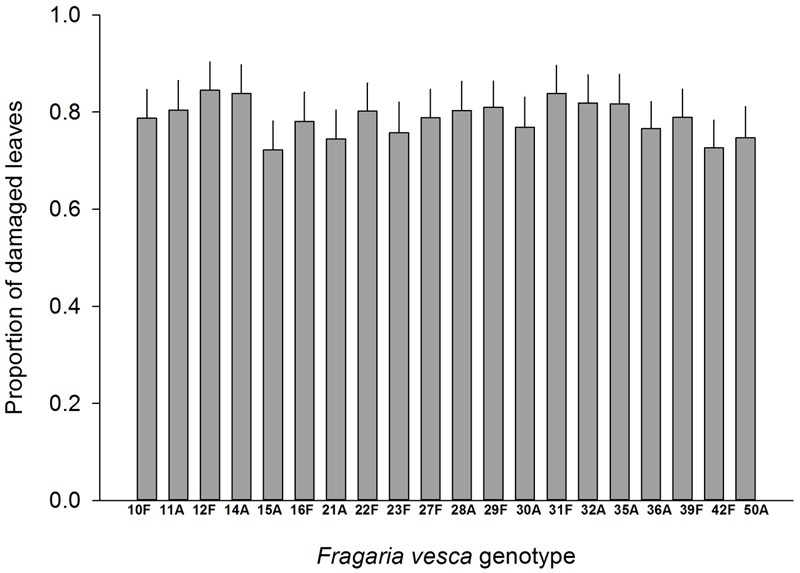
**Variation in resistance of 20 wild woodland strawberry (*F. vesca*) genotypes against *G. sagittariae*.** Resistance was measured as the proportion of leaves damaged, i.e., a lower number of damaged leaves indicates a more resistant plant genotype. Note that ‘Rügen’ cultivars were not included in this experiment. Estimated mean +SE.

**FIGURE 7:**
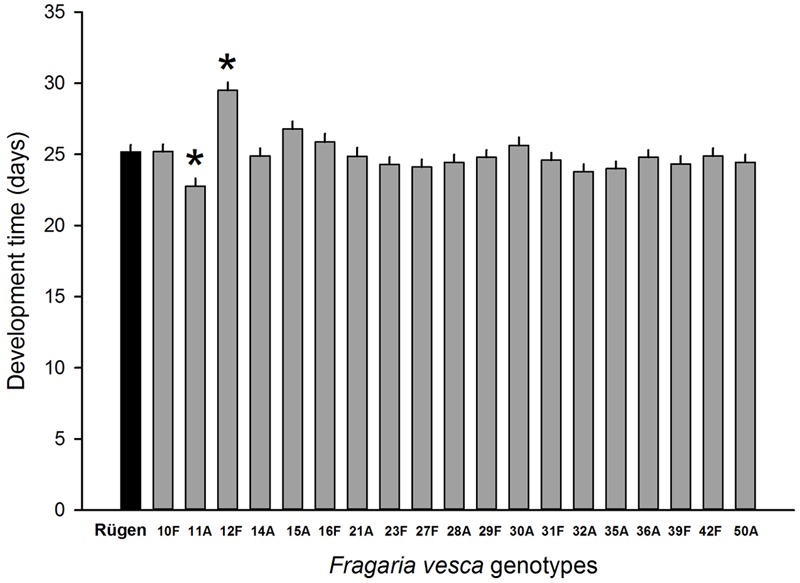
**Variation in resistance of 19 wild woodland strawberry (*F. vesca*) genotypes against *G. tenella*.** Resistance was measured as larval development time from hatching until pupation, i.e., longer development time indicates more resistant plant genotype. Black bar denotes the development time of *G. tenella* on the‘Rügen’ cultivar and gray bars on different wild woodland strawberry genotypes. Asterisks denote statistically significant differences between the development time on a given wild woodland strawberry genotype and the ‘Rügen’ cultivar according to Tukey’s *post hoc* comparisons (*P* < 0.05). Note that wild woodland strawberry genotype 22F was not included in this experiment.

**FIGURE 8:**
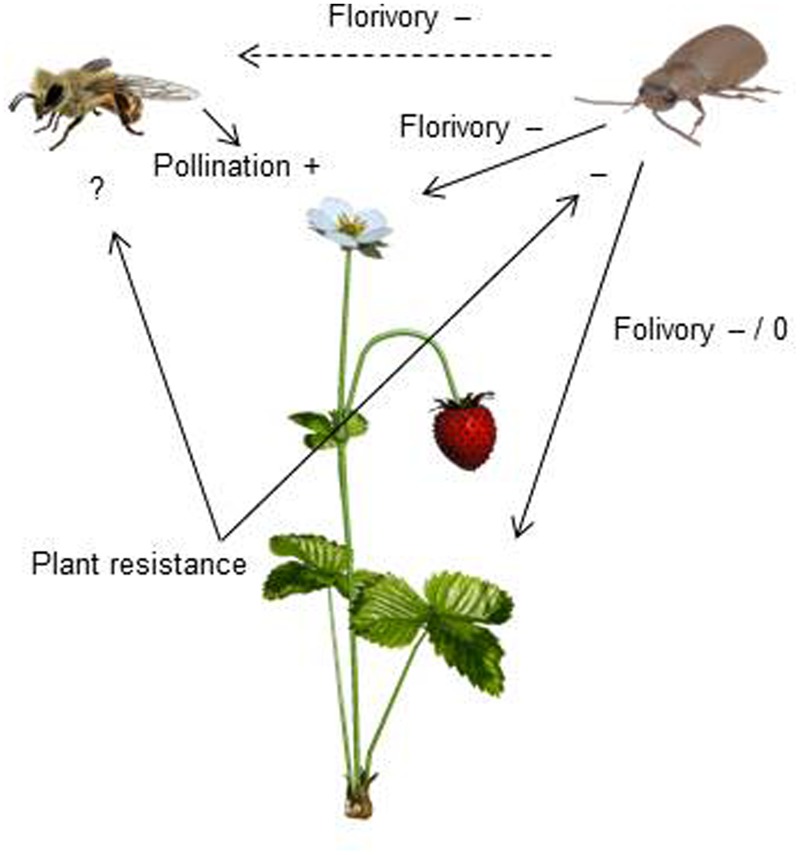
**A conceptual figure presenting how interactions between strawberry, strawberry leaf beetles (SLB) and pollinators affect strawberry yield.** Insect pollination increases (+) strawberry yield. SLB feeding on strawberry flowers has both indirect (dashed arrow) and direct (solid arrow) negative (–) effects on strawberry yield. Indirect effects are due to pollinator discrimination against SLB-damaged flowers. The effect of folivory depends on the amount of damage – low damage has no effect (0) while a higher amount of damage has negative effects (–) on yield (Muola and Stenberg, unpublished data). Resistant plant genotypes are less damaged and herbivore performance is poor on resistant plant genotypes, indicating that plant resistance has a negative (–) effect on SLB. The effect of plant resistance on pollinators remains to be tested (?).

Resistance to SLB varied among wild strawberry genotypes. The proportion of leaves damaged by *G. sagittariae* varied from 0.85 ± 0.06 for the most susceptible plant genotype to 0.72 ± 0.6 for the most resistant plant genotype (χ^2^= 3.1, *d.f.* = 1, *P* = 0.0392), indicating genetic variation in plant resistance (**Figure 6**). In general, smaller plants suffered proportionally more damage than larger plants (*F* = 13.85, *d.f.* = 173, 1, *P* < 0.001; Supplementary Figure S1). However, the effect of plant size on the proportion of damaged leaves was similar for all plant genotypes, indicated by the non-significant genotype by plant size interaction (χ^2^= 0.1, *d.f.* = 1, *P* = 0.376). There was significant variation in development time of *G. tenella* larvae among wild strawberry genotypes (*F* = 5.9, *d.f.* = 19, 164, *P* < 0.001; **Figure 7**). The average larval development time varied from 22.8 ± 1.09 days for the most susceptible plant genotype to 29.5 ± 2.2 days for the least susceptible. *Post hoc* comparisons between the nineteen wild strawberry genotypes and a commercial ‘Rügen’ cultivar indicated that one wild genotype (12F) was significantly more resistant than ‘Rügen’ in terms of development time. The remaining 18 genotypes were either more susceptible (one genotype, 11A) or they possessed intermediate levels of resistance, and did not significantly differ from ‘Rügen’ (**Figure 7**). Our main results on the effects of strawberry-SLB-pollinator interactions on strawberry yield are summarized in **Figure 8**.

## Discussion

### Pollinator Discrimination

Plants with damaged flowers received fewer visits than undamaged flowers, mainly due to hoverfly avoidance rather than bees. Native hoverflies are almost always important pollinators in organic outdoor plantations, as well as in wild strawberry populations, whereas bees are often added to outdoor and indoor cultivations when there is a need to improve pollination services (Albano et al., 2009; Sharma et al., 2014). Thus the reduced visitation by hoverflies could have important impacts on the pollination of damaged flowers in strawberry cultivation. However, given the limited duration of our pollinator observations relating to pollinator discrimination against damaged flowers, the potential differences in discrimination behavior among pollinator groups and their effects on strawberry yield deserve further study. Although we did not investigate the mechanism underlying pollinator discrimination both bees and hoverflies are known to use olfactory as well as visual cues (Gerber and Smith, 1998; Sutherland et al., 1999; Primante and Dötterl, 2010; Klatt et al., 2013), and both these cues are known to be altered by herbivory (Hoffmeister et al., 2016).

### Pollination Success and Yield

We found that SLB-herbivory affected strawberry fruit quality both directly and indirectly via impaired pollination (**Figure 8**). The reduced pollination success for damaged cultivated strawberry adds to a growing body of evidence indicating that diffuse interactions between herbivores and pollinators are likely to be widespread in plants (Kessler et al., 2011; Theis and Adler, 2012; Lucas-Barbosa et al., 2015). Low pollination success of strawberries was associated with smaller fruits and more deformations, both of which are of high economic importance for growers. A previous study, which took reduced fruit size and shape into account, showed that insect pollinators contributed 39% to a total of 2.90 billion US$ made from selling 1.5 million tons of strawberries in the EU in 2009 (Klatt et al., 2014). Thus, in addition to direct costs of herbivory, herbivore-mediated reductions in pollination success could potentially have major economic consequences. It is possible that damaged strawberry plants may be partly able to compensate for the herbivore damage and reduced pollination success by reallocating resources to undamaged flowers or to produce more flowers later in the season when SLB herbivores are less common. The ability to compensate is, however, likely to depend on the amount of damage (Tiffin, 2000). From a Scandinavian grower’s perspective such delayed compensation would have little value as the strawberries are in demand in June, and provide a lower return later in the season. The potential to compensate should nevertheless be investigated further in future studies.

### Potential Solution: Plant Resistance

Our study suggests that even limited herbivore damage has the potential to reduce pollination services as well as yield quantity and quality. An efficient way to combat herbivores is to use pesticides, but the current global trend is to reduce such chemical applications (California Department of Pesticide Regulation [CA DPR], 2015). The recent discovery of the negative effects of neonicotinoids on pollinators (Stanley et al., 2015) may result in a further push for reduced use of many efficient pesticides. Both the direct and indirect effects of herbivory on fruit production require further efforts to develop alternative plant protection strategies, as chemical pesticides disappear from the market. A crucial part of many IPM strategies is resistant cultivars (Eilenberg et al., 2001). Breeding for resistance to herbivores has been neglected during the 20th century as pest problems could be solved with chemical pesticides (Palmgren et al., 2015). Thus, herbivore-resistant cultivars are hard to find for many crops, and domesticated strawberries are among the least resistant and most pesticide-dependent crops (California Department of Pesticide Regulation [CA DPR], 2015). Our screening of SLB resistance in 20 wild woodland strawberry genotypes from SLB’s native area showed the existence of varying levels of resistance against SLB. Furthermore, even though the sampled area represents only a tiny part of the global distribution of woodland strawberry, we found 1 out of 20 strawberry genotypes to be significantly more resistant to SLB than the commonly cultivated cultivar ‘Rügen.’ It is, therefore, likely that even higher resistance might be found with further, more widespread sampling. This finding is important in offering the possibility for reverse breeding to restore high resistance in modern cultivars (Andersen et al., 2015; Palmgren et al., 2015). As the woodland strawberry is diploid, and has a small genome which has already been sequenced (Shulaev et al., 2011), we anticipate that future efforts to identify and breed for SLB resistance could be successful, with potential for application to the octoploid garden strawberry. The multi gene nature of most resistance mechanisms constitutes a challenge for successful breeding. Fortunately, new technologies for targeted genome editing, such as CRISPR/Cas (Fonfara et al., 2016), as well as genomic selection, offer hope that these difficulties can eventually be overcome.

An important question for future research is whether pollination success would increase in parallel with increased resistance. This hope is dependent on sufficiently reduced herbivory, and that plant traits deterring herbivores do not cause trade-offs in resource allocation to traits that attract pollinators (Adler, 2000; Adler et al., 2012). Here, we did not test whether increased resistance improves pollination success in the presence of SLB in wild woodland strawberries. However, the effects of increased resistance on pollination need to be carefully investigated before crops are bred for increased resistance (**Figure 8**).

## Conclusion

Maintaining effective insect pollination services is critical for sustainable intensification of agroecosystems. Although high pollinator abundance is an important factor favored by pesticide-free farming, our findings highlight another important aspect; that pollinators present in a pesticide-free area can refrain from visiting herbivore-damaged crops. The risk of increased herbivory should be taken into account to find new solutions to minimize the negative effects of herbivory on yield and insect pollination when pesticides are phased out. Further studies are needed to explore the potential of breeding for increased herbivore resistance which could be a promising route to reduce damage and increase pollination in strawberries.

## Author Contributions

AM, DW, LM, RG, AP, and JS conceptualized the research and designed the study. AM, DW, and LM conducted the experiments and collected the data. AM analyzed the data with the assistance of LM, DW, and PE. AM and JS wrote the first version of the manuscript. All authors contributed substantially to revisions.

## Conflict of Interest Statement

The authors declare that the research was conducted in the absence of any commercial or financial relationships that could be construed as a potential conflict of interest.
