# Supplementary material for: Direct and Pollinator-Mediated Effects of Herbivory on Strawberry and the Potential for Improved Resistance
Source: Front Plant Sci. 2017 May 18;8:823. doi: 10.3389/fpls.2017.00823 (PMC5435809; doi:10.3389/fpls.2017.00823)
Supplement: Supplementary file 2 [file Image_1.pdf]

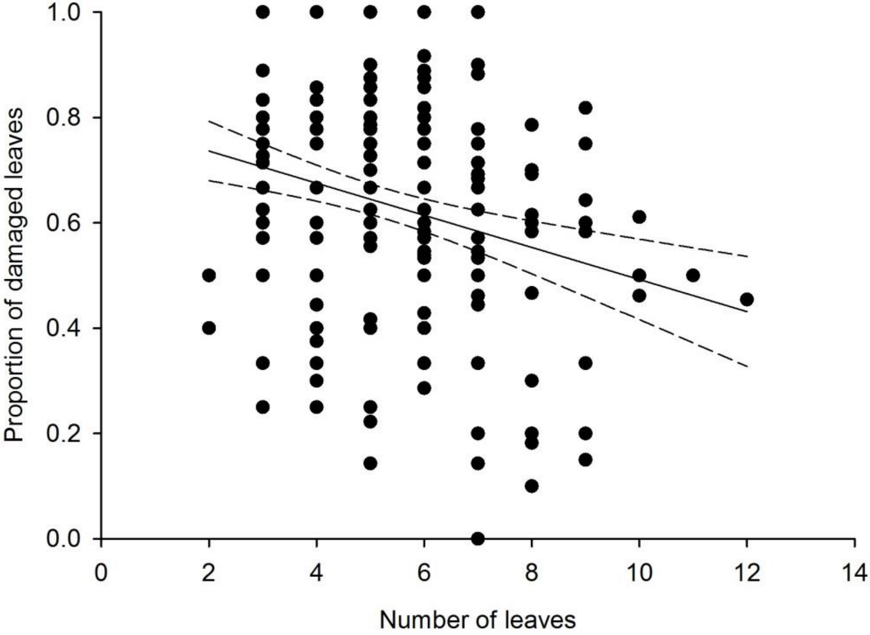

FIGURE S1 | Association between plant size (number of leaves) and proportion of damaged leaves. Proportion of damaged leaves was used as a proxy for plant resistance against *Galerucella sagittariae*. Plant size at the start of the experiment was included as a covariate in the model analysing the genetic variation in plant resistance against *G. sagittariae* to account for the potential effect of plant size for larval feeding. In general, smaller plants had higher proportion of damaged leaves. The effect of plant size was consistent across plant genotypes. Regression line with 95 % confidence interval is given in the figure.
